# β‐Methylphenylalanine exerts neuroprotective effects in a Parkinson's disease model by protecting against tyrosine hydroxylase depletion

**DOI:** 10.1111/jcmm.15571

**Published:** 2020-07-22

**Authors:** Yan Feng, Jianjun Ma, Lipin Yuan

**Affiliations:** ^1^ Department of Neurology Henan Provincial People's Hospital Zhengzhou China

**Keywords:** β‐methylphenylalanine, mitochondrial dysfunction, Parkinson's disease, rotenone, SH‐SY5Y cells

## Abstract

We evaluated the neuroprotective effects of β‐methylphenylalanine in an experimental model of rotenone‐induced Parkinson's disease (PD) in SH‐SY5Y cells and rats. Cells were pre‐treated with rotenone (2.5 µg/mL) for 24 hours followed by β‐methylphenylalanine (1, 10 and 100 mg/L) for 72 hours. Cell viability, reactive oxygen species (ROS) levels, mitochondrial membrane potential (MMP), mitochondrial fragmentation, apoptosis, and mRNA and protein levels of tyrosine hydroxylase were determined. In a rat model of PD, dopamine (DA) and 3,4‐dihydroxyphenylacetic acid (DOPAC) levels, bradykinesia and tyrosine hydroxylase expression were determined. In rotenone–pre‐treated cells, β‐methylphenylalanine significantly increased cell viability and MMP, whereas ROS levels, apoptosis and fragmented mitochondria were reduced. β‐Methylphenylalanine significantly increased the mRNA and protein levels of tyrosine hydroxylase in SH‐SY5Y cells. In the rotenone‐induced rat model of PD, oral administration of β‐methylphenylalanine recovered DA and DOPAC levels and bradykinesia. β‐Methylphenylalanine significantly increased the protein expression of tyrosine hydroxylase in the striatum and substantia nigra of rats. In addition, in silico molecular docking confirmed binding between tyrosine hydroxylase and β‐methylphenylalanine. Our experimental results show neuroprotective effects of β‐methylphenylalanine via the recovery of mitochondrial damage and protection against the depletion of tyrosine hydroxylase. We propose that β‐methylphenylalanine may be useful in the treatment of PD.

## 1 INTRODUCTION

Parkinson's disease (PD) is a well‐known neurodegenerative disease in which the symptoms are a result of increased dopaminergic neuronal cell death.^1^ Subramaniam and Chesselet^2^ reported that mitochondrial dysfunction and reactive oxygen species (ROS) play a major role in the progression of PD. Studies have also reported that PD leads to a reduced level of dopamine (DA), and reduced DA is associated with several adverse clinical motor symptoms, including rigidity, postural instability, bradykinesia and resting tremors.^3^ Chen et al^4^ reported that the dopaminergic neurodegeneration is due to mitochondrial dysfunction. Several studies have reported that PD is a result of mitochondrial dysfunction and the degeneration of dopaminergic neurons. Thus, reduction in mitochondrial damage serves as a therapeutic target for PD.^5^ Rotenone is frequently used to induce PD in animals and cells^6^, ^7^, ^8^ the primary pathological findings, behavioural manifestations and neurochemical features induced by rotenone are similar to those of PD patients. Therefore, the rotenone‐induced rat model of PD is an excellent model to study.^9^

β‐Methylphenylalanine (C_10_H_13_NO_2_) is a non‐proteogenic and non‐natural amino acid with a molecular weight of 179.216 Da. β‐Methylphenylalanine has been shown to exert an antinociceptive effect in experimental animals,^10^ and Ren et al^11^ demonstrated that β‐methylphenylalanine exhibited anti‐arthritic activity in male albino rats. However, limited research is available on the biological effects of β‐methylphenylalanine. In the present study, we analysed the neuroprotective effects of β‐methylphenylalanine in an experimental model of rotenone‐induced PD in SH‐SY5Y cells and rats, and explored whether the neuroprotective effects exerted by β‐methylphenylalanine interfered with the depletion of tyrosine hydroxylase and mitochondrial dysfunction.

## 2 MATERIALS AND METHODS

Dulbecco's modified Eagle's medium, foetal bovine serum and penicillin‐streptomycin were obtained from Sigma‐Aldrich (Shanghai, China). Anti‐tyrosine hydroxylase antibody was purchased from Abcam (ab112; Cambridge, UK). An Annexin V‐FITC Apoptosis Kit (APOAF‐20TST; MilliporeSigma, Burlington, MA, USA) was purchased from MilliporeSigma. Human neuroblastoma SH‐SY5Y cells (subtype: SH‐SY5Y‐A) were purchased from the American Type Culture Collection (Manassas, VA, USA). Retinoic acid was used for the differentiation of SH‐SY5Y cells.

### 2.1 In vitro studies

#### 2.1.1 Sulphorhodamine B assay

Cells were seeded in 96‐well plates (1.5 × 10^4^ cells/well) and pre‐treated with rotenone (2.5 µg/mL) for 24 hours followed by β‐methylphenylalanine (1, 10 and 100 mg/L) for 72 hours. The medium was removed, and the cells were processed for the sulphorhodamine B assay as described previously.^12^ Rasagiline (0.05 mg/L) was used as a positive control.

#### 2.1.2 Determination of intracellular ROS

Cells were seeded in 96‐well plates (1.5 × 10^4^ cells/well) and pre‐treated with rotenone (2.5 µg/mL) for 24 hours followed by β‐methylphenylalanine (1, 10 and 100 mg/L) for 72 hours. Intracellular ROS levels were measured by staining cells with H2DCF‐DA for 30 minutes at 37°C. Fluorescence was visualized using a fluorescence microscope as described previously.^12^

#### 2.1.3 Determination of the mitochondrial membrane potential

Cells were seeded in 6‐well plates (1.5 × 10^4^ cells/well) and pre‐treated with rotenone (2.5 µg/mL) for 24 hours followed by β‐methylphenylalanine (1, 10 and 100 mg/L) for 72 hours. Then, the cells were incubated with rhodamine 123 (2.5 µg/mL) for 60 min in the dark. The mitochondrial membrane potential (MMP) was determined by measuring the fluorescence intensity as described previously.^13^

#### 2.1.4 Determination of mitochondrial fragmentation

Cells were seeded in 6‐well plates (1.5 × 10^4^ cells/well) and pre‐treated with rotenone (2.5 µg/mL) for 24 hours followed by β‐methylphenylalanine (1, 10 and 100 mg/L) for 72 hours. Mitochondrial fragmentation was determined by treating cells with MitoTracker Red for 30 minutes followed by Hoechst 33258 for 15 minutes. Cells were viewed under a fluorescence microscope as described previously.^14^

#### 2.1.5 Reverse transcriptase‐polymerase chain reaction

Cells were seeded in a T75 flask and pre‐treated with rotenone (2.5 µg/mL) for 24 hours followed by β‐methylphenylalanine (1, 10 and 100 mg/L) for 72 hours. Total RNA was extracted from the cells using an RNeasy Mini Kit and transcribed into cDNA using oligo(dT) primers. Reverse transcriptase‐polymerase chain reaction (RT‐PCR) was used to determine tyrosine hydroxylase expression (forward primer: 5′‐TCGGAAGCTGATTGCAGAGA‐3′; reverse primer: 5′‐TTCCGCTGTGTATTCCACATG‐3′). The RT‐PCR mix contained 1 μL of primers, 12.5 μL of RT2 SYBR^®^ Green ROX™ FAST master mix, 10.5 μL of RNase‐free water and 1 μL of cDNA in a final volume of 25 μL. Relative tyrosine hydroxylase expression levels were determined using the 2^−∆∆C^ *^t^* method.^15^

#### 2.1.6 Western blot analysis

Protein expression of tyrosine hydroxylase was determined by Western blotting. Proteins in the cell homogenate were separated by SDS‐PAGE and transferred to nitrocellulose membranes. Then, the membranes were probed with primary rabbit anti‐tyrosine hydroxylase antibodies (1:300 dilution) followed by incubation with a secondary antibody (1:500 dilution) for 60 minutes. Protein levels were determined by densitometry as described previously.^16^

#### 2.1.7 Immunofluorescence

Immunofluorescence was carried out in cells treated with anti‐tyrosine hydroxylase antibodies (1:300 dilution) for 12 hours followed by incubation with FITC‐conjugated secondary antibodies (1:500 dilution; ab6840, Abcam) for 60 minutes. Cells were viewed under a confocal microscope and analysed as described previously.^17^

### 2.2 In vivo studies

#### 2.2.1 Rats

Male albino Wistar strain rats were obtained from the animal house of Henan Provincial People's Hospital, Henan, China. Each group contained six rats, and each rat weighed 190‐210 g. The rats were maintained according to ethical standards for animal welfare.

#### 2.2.2 Rotenone‐induced model of PD

Experimental PD was established according to von Wrangel et al.^18^ Rotenone was dissolved in dimethyl sulphoxide (DMSO) and further diluted in natural oil to a final concentration of 2.5 mg of rotenone/mL (25 μL DMSO/mL). Six rats each were assigned to the treatment and control groups. Rats in the treatment group received rotenone (2.5 mg/kg) once a day by intraperitoneal administration for 60 consecutive days.

#### 2.2.3 Experimental groups

Rats were treated with β‐methylphenylalanine (1, 10 and 100 mg/kg) for 21 days. Rasagiline (0.05 mg/kg) was used as a positive control. Rasagiline is an irreversible inhibitor of monoamine oxidase, which breaks down neurotransmitters such as serotonin, DA and epinephrine/norepinephrine, and is used as a monotherapy in early PD. Control rats received an equal volume of DMSO. All doses were administered orally. The preliminary study was conducted with various concentrations of β‐methylphenylalanine (0.1‐500 mg/kg); optimal and significant effects were observed up to 100 mg/kg. Thus, we tested the concentrations of 1‐100 mg/kg of β‐methylphenylalanine in our subsequent experiments. All procedures involving animals were performed in accordance with the ethical standards of Henan Provincial People's Hospital.

#### 2.2.4 Rotarod test

The rotarod test was performed to determine hindlimb and forelimb balance and co‐ordination. Rats were pre‐trained on the rotating bar of the rotarod unit set on days 10 and 11 before testing on day 12. Three trials were carried out each day during pre‐training, and the rats were kept on the rotating bar for 6 minutes in each trial. Performance was recorded, and the data were subjected to a statistical analysis as described previously.^19^

#### 2.2.5 Measurement of striatal DA and 3,4‐dihydroxyphenylacetic acid levels

High‐performance liquid chromatography (HPLC) was performed to determine the levels of striatal DA and 3,4‐dihydroxyphenylacetic acid (DOPAC). The striatum was dissected, homogenized and centrifuged at 12 000 rpm for 15 minutes at 4°C. The supernatant was collected, filtered with a 0.22‐μm membrane and injected into the HPLC pump. Chromatographic separation was carried out, and the levels of striatal DA and DOPAC were calculated as described previously.^20^

#### 2.2.6 Determination of tyrosine hydroxylase–positive neurons

At the end of the experiment, the rats were anaesthetized with xylazine and ketamine hydrochloride and euthanized by decapitation. Brain tissues were surgically removed, striatum and substantia nigra dissected and fixed in 4% paraformaldehyde. Then, the frozen sections were cut into 5‐μm‐thick sections and incubated with primary rabbit anti‐tyrosine hydroxylase antibodies followed by incubation with secondary antibodies for 60 minutes. Then, tyrosine hydroxylase–immunopositive cells were counted using bright‐field microscopy as described previously.^17^

#### 2.2.7 In silico molecular docking studies

In silico molecular docking was carried out for β‐methylphenylalanine with tyrosine hydroxylase as the target protein. Docking was performed using AutoDock with Cygwin Terminal software, and the binding energy for each active site was determined as described previously.^21^

### 2.3 Statistical analyses

Data are presented as the mean ± standard deviation and were subjected to an analysis of variance. Differences were considered significant at *P* < 0.05.

## 3 RESULTS

### 3.1 In vitro studies

#### 3.1.1 Effect of β‐methylphenylalanine on cell viability and ROS levels

**FIGURE 1:**
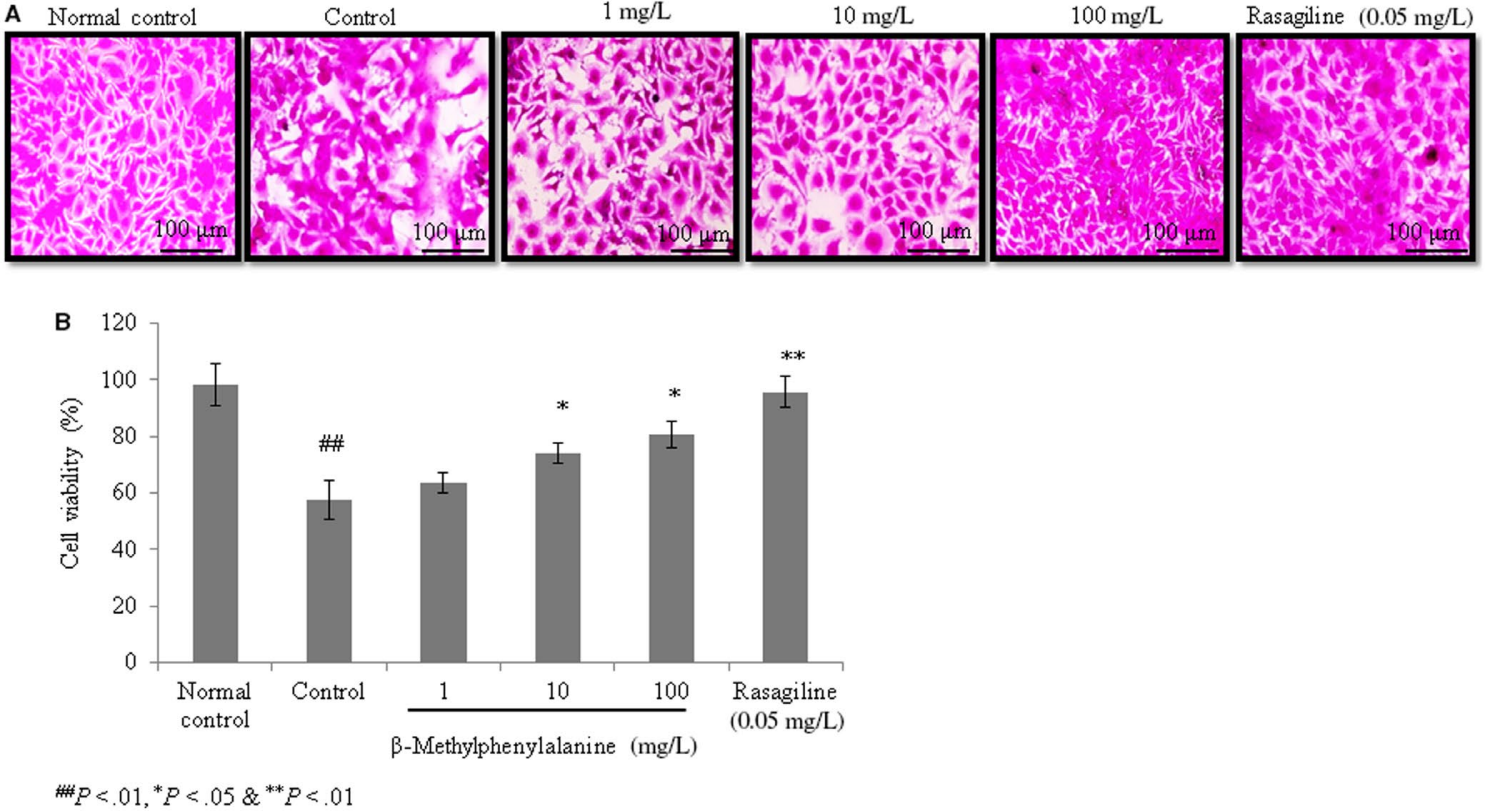
β‐Methylphenylalanine increased the viability of rotenone–pre‐treated cells. Cells were treated with rotenone followed by β‐methylphenylalanine (1‐100 mg/L) for 72 h. A, Microscopic images of viable cells. B, Percentages of viable cells. ^##^ *P* < 0.01 vs normal control. **P < 0.01 and *P < 0.05 vs control. Scale bar, 100 μm

**FIGURE 2:**
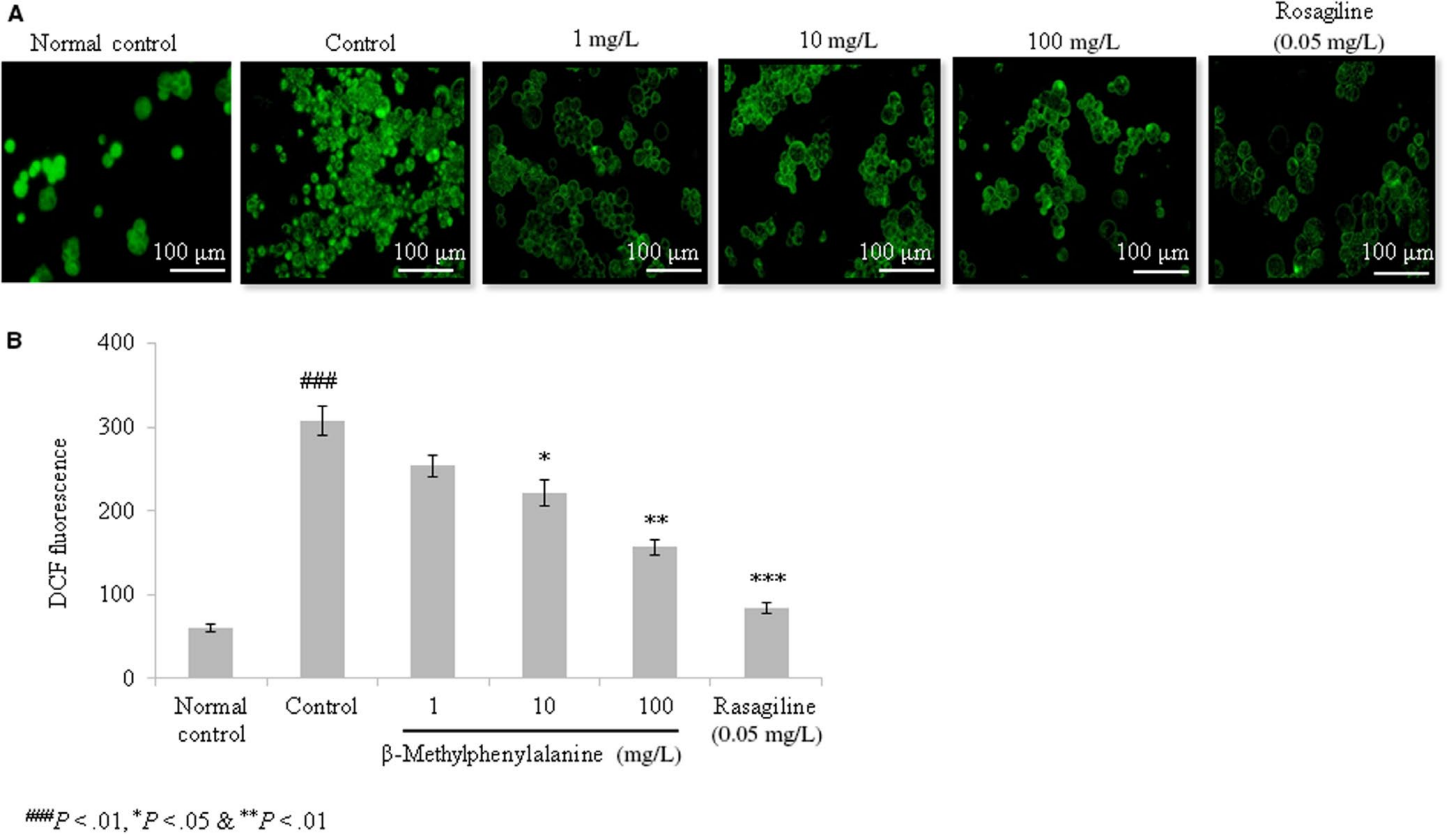
β‐Methylphenylalanine reduced the reactive oxygen species (ROS) levels in rotenone–pre‐treated cells. Cells were treated with rotenone followed by β‐methylphenylalanine (1‐100 mg/L) for 72 h. A, Microscopic images of ROS. B, ROS levels expressed as relative fluorescence units. ^###^ *P* < 0.001 vs normal control. ***P < 0.001, **P < 0.01 and *P < 0.05 vs control. Scale bar, 100 μm

The viability of rotenone–pre‐treated cells was drastically reduced compared with the normal control. However, 1, 10 and 100 mg/L of β‐methylphenylalanine significantly increased cell viability by 10.4%, 29% and 40.2%, respectively (Figure 1A,B, *P* < 0.05). ROS levels were increased in rotenone–pre‐treated cells. However, 1, 10 and 100 mg/L of β‐methylphenylalanine significantly reduced the ROS levels by 17.5%, 28% and 49%, respectively (Figure 2A,B, *P* < 0.05).

#### 3.1.2 Effect of β‐methylphenylalanine on MMP and mitochondrial fragmentation

**FIGURE 3:**
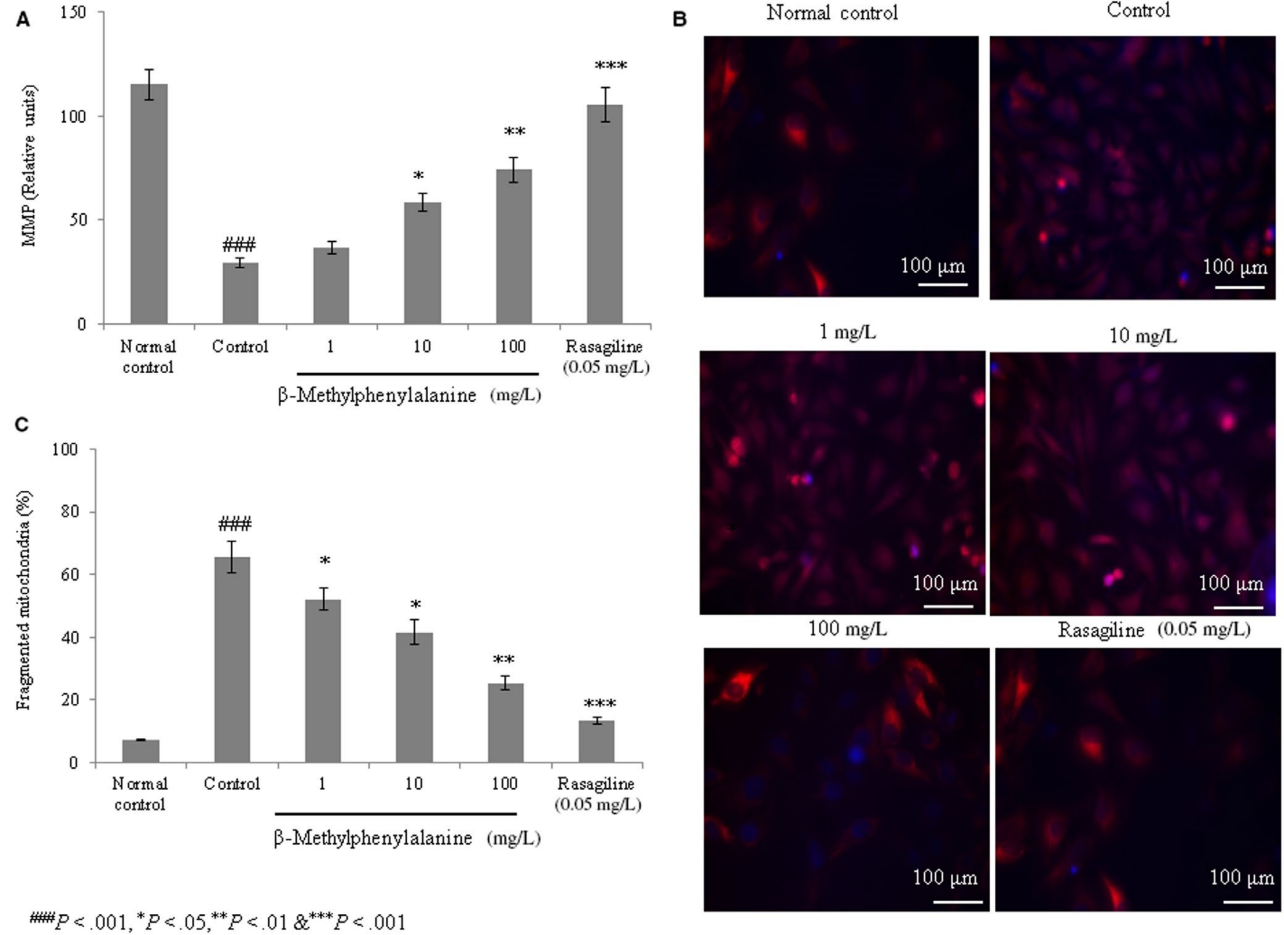
β‐Methylphenylalanine increased the mitochondrial membrane potential (MMP) and mitochondrial fragmentation in rotenone–pre‐treated cells. Cells were treated with rotenone (2.5 µg/mL) for 24 h followed by β‐methylphenylalanine (1‐100 mg/L) for 72 h. A, MMP levels. B, Microscopic images of mitochondrial fragmentation. C, Percentage analysis of mitochondrial fragmentation. ^###^ *P* < 0.001 vs normal control. ***P < 0.001, **P < 0.01 and *P < 0.05 vs control. Scale bar, 100 μm

The MMP was drastically reduced in the control group compared with the normal control. However, 1, 10 and 100 mg/L of β‐methylphenylalanine significantly increased the MMP by 24.9%, 99.6% and 153.2%, respectively (Figure 3A, *P* < 0.05). The number of fragmented mitochondria, an indicator of mitochondrial dysfunction, was increased in rotenone–pre‐treated cells. However, 1, 10 and 100 mg/L of β‐methylphenylalanine significantly reduced the number of fragmented mitochondria by 20.7%, 36.6% and 61.5%, respectively (Figure 3B,C, *P* < 0.05).

#### 3.1.3 Effect of β‐methylphenylalanine on apoptosis

**FIGURE 4:**
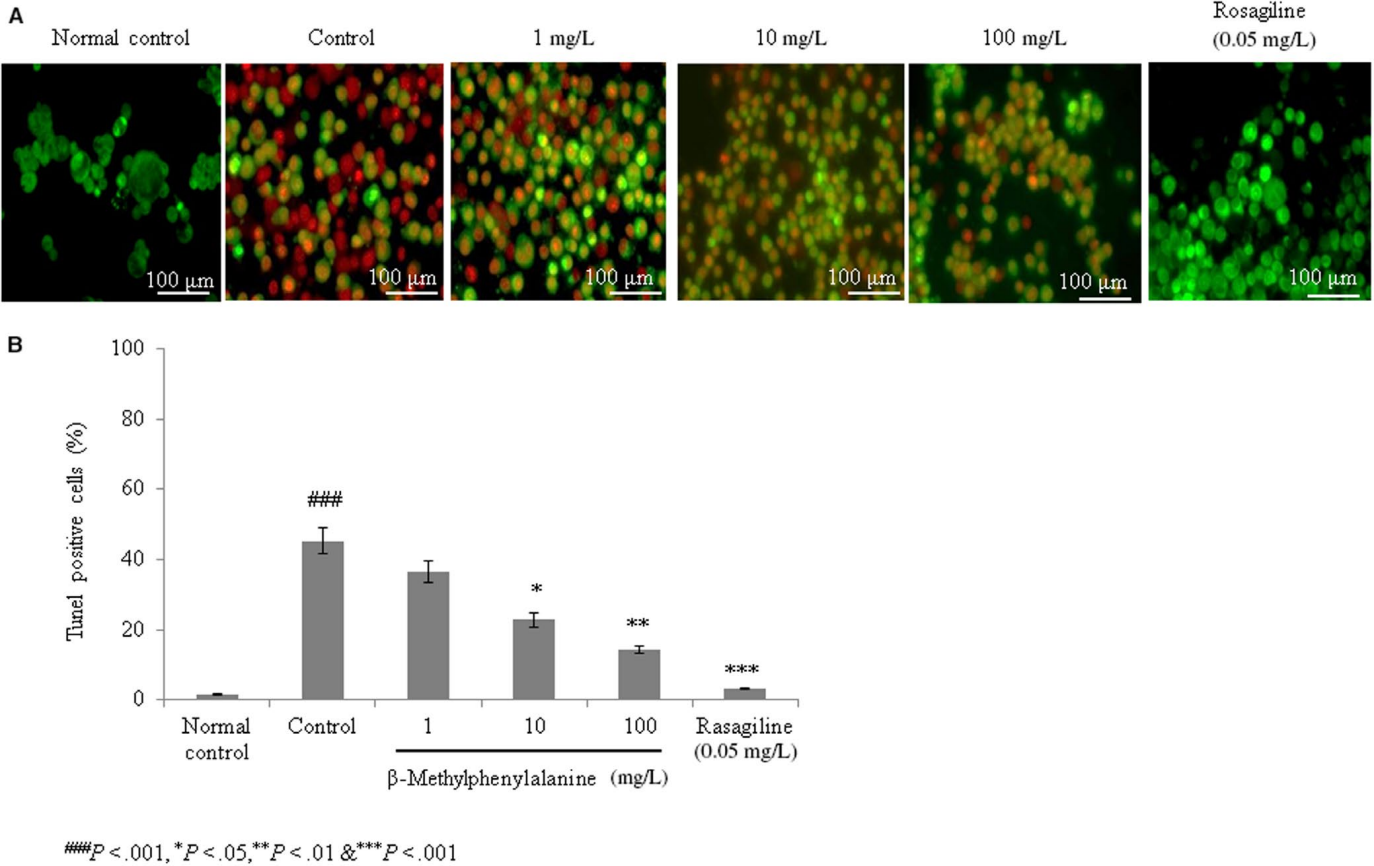
β‐Methylphenylalanine decreased apoptosis in rotenone–pre‐treated cells. Cells were treated with rotenone followed by β‐methylphenylalanine (1‐100 mg/L) for 72 h. A, Microscopic images of apoptosis. B, Percentages of apoptotic cells. ^###^ *P* < 0.001 vs normal control. ***P < 0.001, **P < 0.01 and *P < 0.05 vs control. Scale bar, 100 μm

The frequency of apoptosis was higher in rotenone–pre‐treated SH‐SY5Y cells (control) compared with the normal control. However, 1, 10 and 100 mg/L of β‐methylphenylalanine significantly reduced the frequency of apoptosis by 19.2%, 49.7% and 68.6%, respectively (Figure 4A,B, *P* < 0.05).

#### 3.1.4 Effect of β‐methylphenylalanine on the expression of tyrosine hydroxylase

**FIGURE 5:**
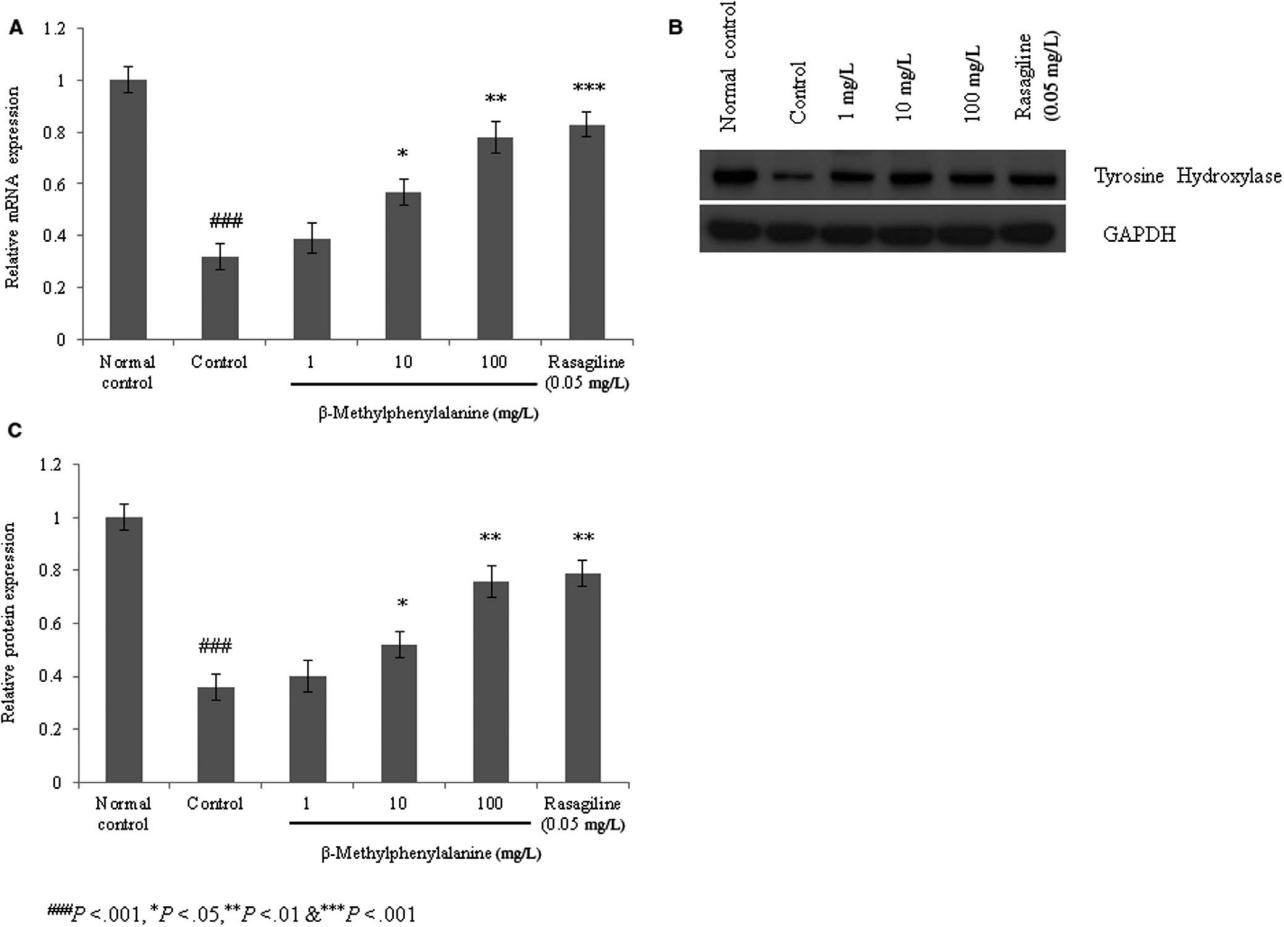
β‐Methylphenylalanine increased the mRNA and protein levels of tyrosine hydroxylase in rotenone–pre‐treated SH‐SY5Y cells. Cells were treated with rotenone followed by β‐methylphenylalanine (1‐100 mg/L) for 72 h. A, The mRNA levels of tyrosine hydroxylase. B, Western blotting for tyrosine hydroxylase. C, Densitometry analysis of B. ^###^ *P* < 0.001 vs normal control. ***P < 0.001, **P < 0.01 and *P < 0.05 vs control

**FIGURE 6:**
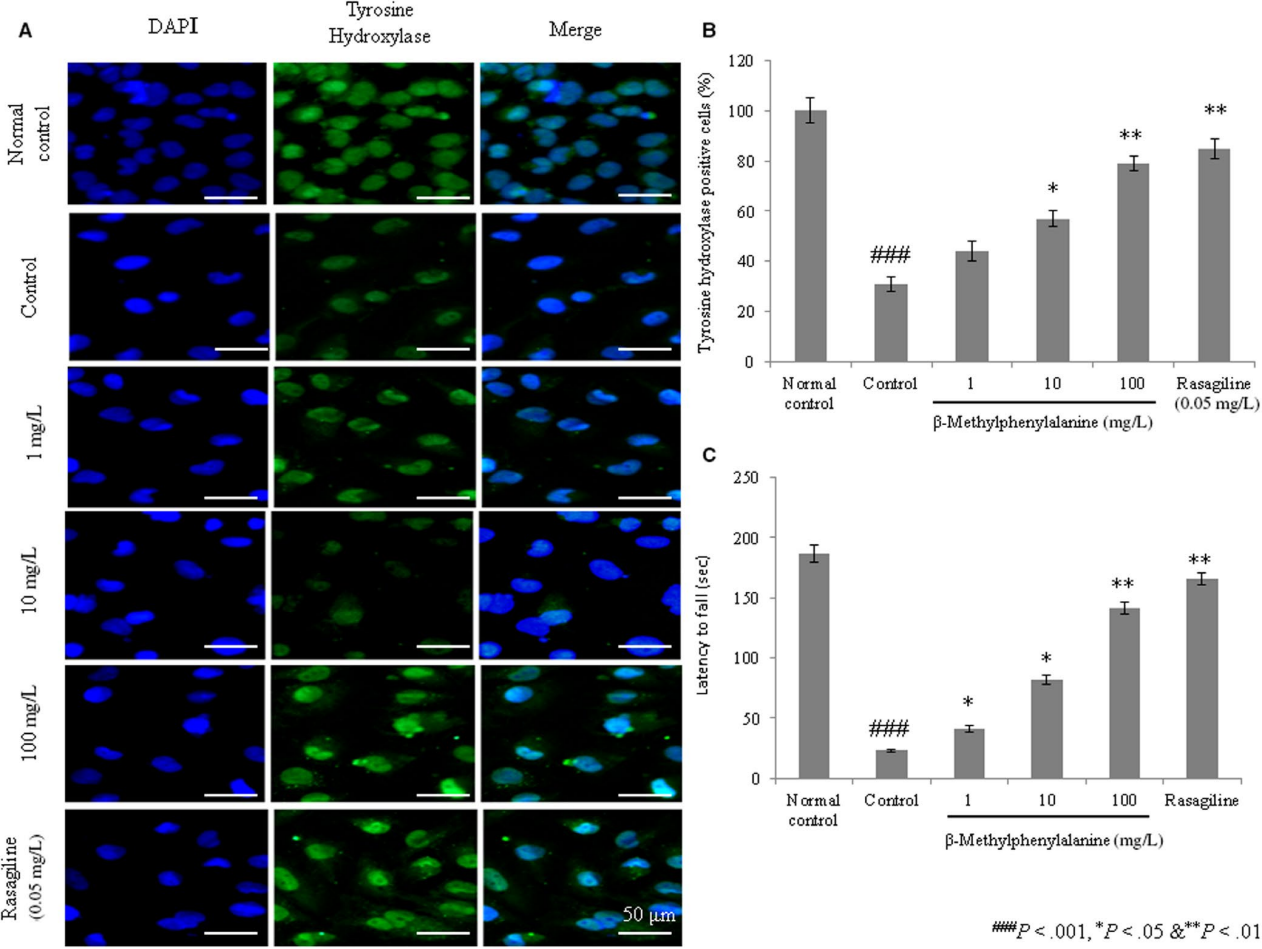
β‐Methylphenylalanine increased the protein levels of tyrosine hydroxylase in rotenone–pre‐treated cells as determined by immunofluorescence. Cells were treated with rotenone followed by β‐methylphenylalanine (1‐100 mg/L) for 72 h. A, Immunohistochemical staining for tyrosine hydroxylase. B, Tyrosine hydroxylase–positive cells. C, Effects of β‐methylphenylalanine on behavioural co‐ordination deficiencies in rotenone‐treated experimental PD model rats. ^###^ *P* < 0.001 vs normal control. ***P < 0.001, **P < 0.01 and *P < 0.05 vs control. Scale bar, 50 μm

The mRNA levels of tyrosine hydroxylase were decreased in rotenone–pre‐treated cells compared with the normal group. However, 1, 10 and 100 mg/L of β‐methylphenylalanine significantly increased the mRNA levels of tyrosine hydroxylase by 21%, 78% and 143.7%, respectively (Figure 5A, *P* < 0.05). Protein expression of tyrosine hydroxylase was increased by 11.1%, 44.4% and 111.1% with 1, 10 and 100 mg/L of β‐methylphenylalanine, respectively (Figure 5B,C, *P* < 0.05). Immunofluorescence confirmed increased expression of tyrosine hydroxylase with increases of 9%, 31% and 54% following treatment with 1, 10 and 100 mg/L of β‐methylphenylalanine, respectively (Figure 6A,B, *P* < 0.05).

### 3.2 In vivo studies

#### 3.2.1 Effect of β‐methylphenylalanine on behavioural co‐ordination

The in vivo effect of β‐methylphenylalanine was evaluated using rotenone‐treated PD model rats. The rotarod test was conducted with a minor modification to evaluate whether β‐methylphenylalanine protects against the motor deficit induced by rotenone neurotoxicity. Rats administered rotenone showed a substantial reduction in latency compared with normal rats (Figure 6C, *P* < 0.05). The latent period was markedly increased by 78%, 256.5% and 517.4% with 1, 10 and 100 mg/kg of β‐methylphenylalanine, respectively (Figure 6C, *P* < 0.05). Thus, β‐methylphenylalanine effectively inhibited rotenone‐induced behavioural co‐ordination deficiencies.

#### 3.2.2 Effect of β‐methylphenylalanine on the expression of tyrosine hydroxylase

**FIGURE 7:**
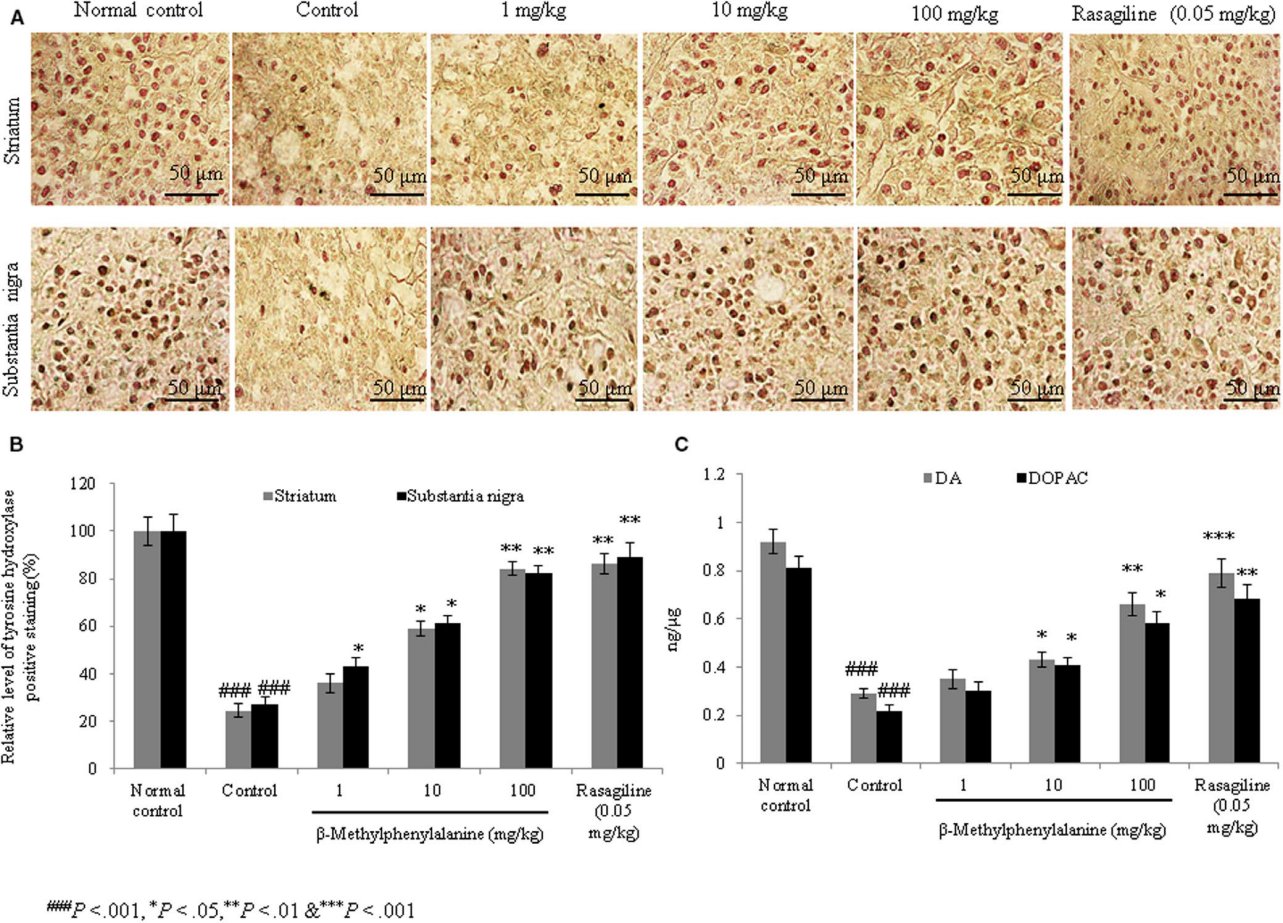
Effect of β‐methylphenylalanine on tyrosine hydroxylase expression in the striatum and substantia nigra of rotenone‐treated experimental PD model rats. Rats were treated with rotenone followed by β‐methylphenylalanine (1, 10 and 100 mg/kg) for 21 d. A, Immunohistochemical staining for tyrosine hydroxylase. B, Percentage analysis of A. C, Dopamine and 3,4‐dihydroxyphenylacetic acid (DOPAC) levels in the striatum of control and treated rats. ^###^ *P* < 0.001 vs normal control. ***P < 0.001, **P < 0.01 and *P < 0.05 vs control. Scale bar, 50 μm

Immunohistochemistry revealed that tyrosine hydroxylase expression was reduced by 75.5% and 72.6% in the striatum and substantia nigra of control rats compared with the normal group. However, 1, 10 and 100 mg/kg of β‐methylphenylalanine increased the expression of tyrosine hydroxylase in the striatum by 47.8%, 141.2% and 244.1%, respectively (Figure 7A,B, *P* < 0.05), whereas in the substantia nigra, expression was increased by 56.2%, 124.5% and 201% at concentrations of 1, 10 and 100 mg/kg, respectively (Figure 7A,B, *P* < 0.05).

#### 3.2.3 Effect of β‐methylphenylalanine on DA and DOPAC levels

Rats administered rotenone showed a substantial reduction in the levels of DA and DOPAC compared with normal rats (Figure 7C, *P* < 0.05). However, 1, 10 and 100 mg/kg of β‐methylphenylalanine significantly increased DA levels by 28%, 72% and 132%, respectively (*P* < 0.05), whereas DOPAC levels were increased by 58%, 141.2% and 276.5% at 1, 10 and 100 mg/kg, respectively (Figure 7C, *P* < 0.05). DOPAC/DA ratio was significantly increased to the near‐normal range.

#### 3.2.4 In silico molecular docking study

In silico molecular docking confirmed the binding of tyrosine hydroxylase and β‐methylphenylalanine. The binding affinity of β‐methylphenylalanine for the tyrosine hydroxylase subunit was confirmed by in silico docking; the glide energy was −8.55 kcal/mol (Table S1).

## 4 DISCUSSION

We analysed the neuroprotective effects of β‐methylphenylalanine, which occurred via alleviation of mitochondrial damage and prevention of tyrosine hydroxylase depletion, in a rotenone‐induced model of PD in SH‐SY5Y cells and rats. As mitochondria are involved in the pathology of PD, we propose that β‐methylphenylalanine may be a potent therapeutic agent in the treatment of PD. Alzheimer's disease, dementia and PD have multifactorial patho‐etiological origins. Mitochondrial dysfunction and oxidative stress are major pathophysiological mechanisms involved in neurodegeneration.^22^ Thus, any drug or agent that prevents oxidative stress and mitochondrial dysfunction could serve as an effective anti‐neurodegenerative therapeutic agent.^5^ Creatine and CoQ10 have been shown to exhibit therapeutic effects against neurodegenerative diseases such as PD. However, there are insufficient data available to support the use of creatine and CoQ10 in the treatment of PD.^23^, ^24^ Therefore, a continued search for novel therapeutic agents against neurodegenerative diseases is necessary.

Rotenone induces a substantial loss of dopaminergic neurons in the nigrostriatal region, which plays a critical role in motor function. We found that β‐methylphenylalanine treatment significantly recovered behavioural deficits and dopaminergic neuronal death. β‐Methylphenylalanine also reduced the depletion of striatal DA in rotenone‐treated rats due to the rescue of dopaminergic neurons. Our results reveal that β‐methylphenylalanine rescued rotenone‐induced mitochondrial dysfunction, including MMP, mitochondrial fragmentation and intracellular ROS levels, in SH‐S5Y5 cells and a rat model of PD.

Tyrosine hydroxylase is an important enzyme that plays a major role in L‐DOPA formation, which is the initial, rate‐limiting step in DA biosynthesis; thus, PD and tyrosine hydroxylase are directly connected.^25^ L‐DOPA is a precursor required for DA synthesis, and DA is a precursor required for the synthesis of norepinephrine and epinephrine. Thus, a defect in the biosynthesis of norepinephrine and epinephrine is crucial for the progression of PD and other neurodegenerative disorders.^26^ Choi et al^27^ reported the neuroprotective effects of herbal ethanol extracts of *Gynostemma pentaphyllum*; these extracts prevented the loss of tyrosine hydroxylase in a rat model of PD. Li and Pu^28^ reported neuroprotective effects of kaempferol, which prevented the loss of tyrosine hydroxylase in a 1‐methyl‐4‐phenyl‐1,2,3,6‐tetrahydropyridine‐induced mouse model of PD. Ablat et al^29^ reported the neuroprotective potential of flavonoid extracts of safflower, which also prevented the loss of tyrosine hydroxylase in a rotenone‐induced rat model. In this study, β‐methylphenylalanine prevented the rotenone‐induced loss of tyrosine hydroxylase in SH‐S5Y5 cells and a rat model of PD.

## 5 CONCLUSION

Taken together, our experimental results show the neuroprotective effects of β‐methylphenylalanine, which occurred via the recovery of mitochondrial dysfunction and prevention of tyrosine hydroxylase depletion in rotenone‐induced PD in SH‐SY5Y cells and rats.

## CONFLICT OF INTEREST

The authors declare that they have no conflict of interest.

## AUTHORS' CONTRIBUTIONS

YF and JM conducted experiments and collected data. LY carried out data interpretation, review of literature and manuscript drafting.

## CONSENT TO PARTICIPATE

Not applicable.

## ETHICS APPROVAL

All animal experiments were approved by the ethical committee of Department of Neurology, Henan Provincial People's Hospital, No 7 of Weiwu Road, Zhengzhou, Henan 450003, China.

## CONSENT FOR PUBLICATION

Not applicable.

## Supporting information

Table S1

## Data Availability

The corresponding author could provide all the experimental data on valid request.

## References

[1] Creed RB , Goldberg MS . New developments in genetic rat models of Parkinson's disease. Mov Disord. 2018;33(5):717‐729. 29418019 10.1002/mds.27296 PMC5992003

[2] Subramaniam SR , Chesselet MF . Mitochondrial dysfunction and oxidative stress in Parkinson's disease. Prog Neurogibol. 2013;107:17‐32. 10.1016/j.pneurobio.2013.04.004 PMC3742021 23643800

[3] Dauer W , Przedborski S . Parkinson's disease: mechanisms and models. Neuron. 2013;39:889‐909. 10.1016/s0896-6273(03)00568-3 12971891

[4] Chen C , Turnbull DM , Reeve AK . Mitochondrial dysfunction in Parkinson's disease‐cause or consequence? Biology. 2019;8(2):38. 10.3390/biology8020038 PMC6627981 31083583

[5] Pak YK , Jeong JH . Mitochondria: the secret chamber of therapeutic targets for age‐associated degenerative diseases. Biomol Therap. 2010;18:235‐245.

[6] Zhao XL , Gu ZL , Qin ZH . Chronic subcutaneous injection of rotenone produces rodent Parkinsonian models. Zhongguo Yaolixue Tongbao. 2005;21(10):1274‐1277.

[7] Chen X , Zhang N , Zhao H . The relations of behavioral features and loss of Nigrostriatum on the model of Parkinson's disease induced by rotenone on rats. Zhongguo Shenjing Jingshen Jibing Zazhi. 2008;34(4):232‐234.

[8] Li C , Chen X , Zhang N . Changes of expression of apoptosis‐related proteins Bcl‐2 and Bax in Parkinson's disease rat induced by rotenone. Zhongguo Shiyan Dongwu Xuebao. 2009;17(1):50‐52.

[9] Filomeni G , Graziani I , De Zio D , et al. Neuroprotection of kaempferol by autophagy in models of rotenone‐mediated acute toxicity: possible implications for Parkinson's disease. Neurobiol Aging. 2012;33(4):767‐785. 20594614 10.1016/j.neurobiolaging.2010.05.021

[10] Kovács G , Petrovszki Z , Mallareddy JR , Tóth G , Benedek G , Horváth G . Characterization of antinociceptive potency of endomorphin‐2 derivatives with unnatural amino acids in rats. Acta Physiol Hung. 2012;99:353‐363. 22982723 10.1556/APhysiol.99.2012.3.12

[11] Ren SX , Zhang B , Lin Y , Ma DS , Li H . Mechanistic evaluation of anti‐arthritic activity of β‐methylphenylalanine in experimental rats. Biomed Pharmacother. 2019;113:108730. 30861411 10.1016/j.biopha.2019.108730

[12] Pandurangan M , Enkhtaivan G , Mistry B , Patel RV , Moon S , Kim DH . β‐Alanine metabolic recovery for the amelioration of human cervical and renal tumors. Amino Acids. 2017;49:1373‐1380. 28516269 10.1007/s00726-017-2437-y

[13] Joshi DC , Bakowska JC . Determination of mitochondrial membrane potential and reactive oxygen species in live rat cortical neurons. J Vis Exp. 2011;23(51):2704. 10.3791/2704 PMC3143685 21654619

[14] Martorell‐Riera A , Segarra‐Mondejar M , Reina M , Martínez‐Estrada OM , Soriano FX . Mitochondrial fragmentation in excitotoxicity requires ROCK activation. Cell Cycle. 2015;14(9):1365‐1369. 25789413 10.1080/15384101.2015.1022698 PMC4612563

[15] Liss B . Improved quantitative real‐time RT‐PCR for expression profiling of individual cells. Nucleic Acids Res. 2002;30(17):e89. 12202777 10.1093/nar/gnf088 PMC137434

[16] Dmitriev AD , Factor MI , Segal OL , et al. Western blot analysis of human and rat serotonin transporter in platelets and brain using site‐specific antibodies: evidence that transporter undergoes endoproteolytic cleavage. Clin Chim Acta. 2005;356(1‐2):76‐94. 15936305 10.1016/j.cccn.2004.12.019

[17] Allaire A , Picard‐Jean F , Bisaillon M . Immunofluorescence to monitor the cellular uptake of human lactoferrin and its associated antiviral activity against hepatitis C virus. J Vis Exp. 2015;104:53053. 10.3791/53053 PMC4692635 26485289

[18] von Wrangel C , Schwabe K , John N , Krauss JK , Alam M . The rotenone‐induced rat model of Parkinson's disease: behavioral and electrophysiological findings. Behav Brain Res. 2015;279:52‐61. 25446762 10.1016/j.bbr.2014.11.002

[19] Gorgone G . Coenzyme Q10, hyperhomocysteinemia and MTHFR C677T polymorphism in levodopa‐treated Parkinson's disease patients. NeuroMol Med. 2012;14:84‐90. 10.1007/s12017-012-8174-1 22354693

[20] Kim HG . Effects of the root bark of Paeonia suffruticosa on mitochondria‐mediated neuroprotection in an MPTP‐induced model of Parkinson's disease. Food Chem Toxicol. 2014;65:293‐300. 24389454 10.1016/j.fct.2013.12.037

[21] Jamal MS , Parveen S , Beg MA , et al. Anticancer compound plumbagin and its molecular targets: a structural insight into the inhibitory mechanisms using computational approaches. PLoS ONE. 2014;9(2):e87309. 24586269 10.1371/journal.pone.0087309 PMC3937309

[22] Gotz ME . Altered redox state of platelet coenzyme Q10 in Parkinson's disease. J Neural Transm. 2010;107:41‐48. 10.1007/s007020050003 10809402

[23] Beal MF . Neuroprotective effects of creatine. Amino Acids. 2011;40:1305‐1313. 21448659 10.1007/s00726-011-0851-0

[24] Zhu ZG . The efficacy and safety of coenzyme Q10 in Parkinson's disease: a meta‐analysis of randomized controlled trials. Neurological Sci. 2017;38:215‐224. 10.1007/s10072-016-2757-9 27830343

[25] Tabrez S , Jabir NR , Shakil S . A synopsis on the role of tyrosine hydroxylase in Parkinson's disease. CNS Neurol Disord Drug Targets. 2012;11(4):395‐409. 22483313 10.2174/187152712800792785 PMC4978221

[26] Blanchard‐Fillion B , Souza JM , Friel T , et al. Nitration and inactivation of tyrosine hydroxylase by peroxynitrite. J Biol Chem. 2001;276(49):46017‐46023. 11590168 10.1074/jbc.M105564200

[27] Choi HS , Park MS , Kim SH , Hwang BY , Lee CK , Lee MK . Neuroprotective effects of herbal ethanol extracts from Gynostemma pentaphyllum in the 6‐hydroxydopamine‐lesioned rat model of Parkinson's disease. Molecules. 2010;15(4):2814‐2824. 20428081 10.3390/molecules15042814 PMC6257318

[28] Li S , Pu XP . Neuroprotective effect of kaempferol against a 1‐methyl‐4‐phenyl‐1,2,3,6‐tetrahydropyridine‐induced mouse model of Parkinson's disease. Biol Pharm Bull. 2011;34(8):1291‐1296. 21804220 10.1248/bpb.34.1291

[29] Ablat N , Lv D , Ren R , et al. Neuroprotective effects of a standardized flavonoid extract from safflower against a rotenone‐induced rat model of Parkinson's disease. Molecules. 2016;21(9):1107. 10.3390/molecules21091107 PMC6274364 27563865

