# Supplementary material for: β‐Methylphenylalanine exerts neuroprotective effects in a Parkinson's disease model by protecting against tyrosine hydroxylase depletion
Source: J Cell Mol Med. 2020 Jul 22;24(17):9871–80. doi: 10.1111/jcmm.15571 (PMC7520294; doi:10.1111/jcmm.15571)
Supplement: Supplementary file 1 — Table S1 [file JCMM-24-9871-s001.docx]

Supplementation of β-methylphenylalanine exert neuroprotective effect in SH‑SY5Y cell and rat model of Parkinson disease by recovering the mitochondrial dysfunction and protecting the depletion of tyrosine hydroxylase

Yan Feng*, Jianjun Ma and Lipin Yuan

Department of Neurology, Henan provincial people’s hospital, Zhengzhou, Henan, 450003, China

Running title: β-Methylphenylalanine and Parkinson’s disease

*Corresponding author: Yan Feng,

Department of Neurology, Henan provincial people’s hospital,

No 7 of Weiwu Road, Zhengzhou, Henan, 450003, China

Tel/Fax:  [+86 371 6558 0014](https://www.google.com/search?safe=active&ei=DY_BXLy_Kozr-QaS7rbICA&q=%E6%B2%B3%E5%8D%97%E7%9C%81%E4%BA%BA%E6%B0%91%E5%8C%BB%E9%99%A2&oq=%E6%B2%B3%E5%8D%97%E7%9C%81%E4%BA%BA%E6%B0%91%E5%8C%BB%E9%99%A2&gs_l=psy-ab.3..35i39j0i30l9.146461.146461..146682...0.0..0.85.85.1......0....1..gws-wiz.MNFQX0LS4pk)

Email: [yanf22514@gmail.com](mailto:yanf22514@gmail.com)

**Table 1S**: Binding score of five different active site of tyrosine hydroxylase with β-methylphenylalanine

| S.No | Cluster Rank | Binding Residual Amino acid | Run | Minimum binding energy (kcal/mol) | Reference RMSD |
| --- | --- | --- | --- | --- | --- |
| 1 | 4 | Leu 525 | 9 | -7.77 | 68.64 |
| 2 | 2 | Phe 404 | 5 | -8.12 | 66.87 |
| 3 | 5 | Leu 384 | 11 | -7.75 | 64.25 |
| 4 | 1 | Leu 387 | 7 | -8.82 | 65.95 |
| 5 | 3 | Leu 346 | 5 | -7.85 | 66.74 |
